# ﻿A taxonomic revision of the Old World genus *Dichoteleas* Kieffer (Hymenoptera, Scelionidae)

**DOI:** 10.3897/zookeys.1182.104943

**Published:** 2023-10-13

**Authors:** Johanna Schwartz, Simon Van Noort, Norman F. Johnson

**Affiliations:** 1 Department of Entomology, The Ohio State University, Columbus, Ohio, USA; 2 Research and Exhibitions Department, South African Museum, Iziko Museums of South Africa, Cape Town 8000, South Africa; 3 Department of Biological Sciences, University of Cape Town, Private Bag, Rondebosch, 7701, South Africa; 4 Department of Evolution, Ecology & Organismal Biology, The Ohio State University, 1315 Kinnear Road, Columbus, Ohio 43212, USA

**Keywords:** Egg-parasitoid, Platygastroidea, revision, Scelioninae, taxonomy, tropical

## Abstract

The genus *Dichoteleas* Kieffer (Scelionidae: Scelioninae) is known only from the Old World: Kenya, Tanzania, Malawi, South Africa, Madagascar, southern India, the island of New Guinea, and eastern Australia. After revision, 10 species are recognized. Four species were previously recognized and are redescribed: *D.ambositrae* Risbec (Madagascar), *D.indicus* Saraswat (India: Kerala), *D.rugosus* Kieffer (Australia: Queensland), and *D.subcoeruleus* Dodd (Australia: Queensland). Six species are described as new: *D.fulgidus***sp. nov.** (Indonesia: Papua Barat), *D.fuscus***sp. nov.** (Papua New Guinea, Australia: Queensland), *D.hamatus***sp. nov.** (Kenya, Tanzania, Malawi, South Africa)., *D.rubyae***sp. nov.** (Madagascar), *D.striatus***sp. nov.** (Madagascar), and *D.umbra***sp. nov.** (Tanzania). *Dichoteleaspappi* Szabó is treated as a junior synonym of *D.rugosus.* An identification key to species of the genus is provided.

## ﻿Introduction

The genus *Dichoteleas* was first described by Jean-Jacques Kieffer in 1907 on the basis of a single male specimen collected in Mackay, North Queensland, Australia. It was distinguished from *Pentacantha* Ashmead (a genus of the subfamily Teleasinae) by the “Thorax mit drei spitzen Zähnen” (thorax with three pointed teeth) and the presence of the postmarginal vein on the forewing (Kieffer, 1907). Kieffer did not specify in his generic description on which parts of the mesosoma these teeth occur, but in his description of the sole species, *D.rugosus*, he indicated that the teeth are found on the sides of the mesoscutellum and medially on the metanotum. After collecting a female of the type species, Dodd (1926) added that the antennal club had 7 segments. Later, Masner (1976) proposed that *Dichoteleas* could be identified by its large hairless eyes, elongate maxillary palpi, and subtridentate mandibles.

In the years since its description, five species have been described in the genus. Three were described from Australia (*D.rugosus* Kieffer, *D.subcoeruleus* Dodd, and *D.pappi* Szabó), one from Madagascar (*D.ambositrae* Risbec), and one from India (*D.indicus* Saraswat). Only *D.subcoeruleus* was described based on more than a single sex, and none of these were based on more than five specimens. In their revision of Australian Scelioninae, Galloway and Austin (1984) noted that *D.pappi* may be a junior synonym of *D.rugosus*, since the species have similar ranges, and *Dichoteleaspappi* was described from one female specimen, while *D.rugosus* was described from a male.

Masner (1976) placed *Dichoteleas* in the tribe Calliscelionini of the subfamily Scelioninae, although he mentioned that the genus was “difficult to classify tribally.” He also cited a possible relationship with *Amblyscelio* Kieffer or *Neoscelio* Dodd. *Dichoteleas* was grouped with *Amblyscelio* and *Oxyteleia* Kieffer In the 4-gene analysis of Chen et al. (2021), but the bootstrap support for this was relatively weak. *Dichoteleas* is fairly unusual among platygastroids in that some of the species are distinctly metallic in color. To the best of our knowledge, all members of the subfamily Scelioninae are egg parasitoids of spiders or other insects, but there are currently no host records for *Dichoteleas* and little else is known of this group.

The goals of this paper are to incorporate information from newly collected specimens, revise the circumscription of described species on the basis of these new data, document and describe hypothesized new species within the genus, and to provide a comprehensive identification key for the species of *Dichoteleas*.

## ﻿Methods

This work is based on specimens from the Australian National Insect Collection (**ANIC**; Canberra, Australia), Bernice P. Bishop Museum (**BPBM**; Honolulu, HI), California Academy of Sciences (**CAS**; San Francisco, CA), Canadian National Collection of Insects (**CNCI**; Ottawa, Canada), C.A. Triplehorn Insect Collection (**OSUC**; The Ohio State University, Columbus, OH), Hungarian Natural History Museum (**HNHM**; Budapest, Hungary), International Centre of Insect Physiology and Ecology (**ICIPE**, Nairobi, Kenya), Muséum National d’Histoire Naturelle (**MNHN**; Paris, France), ﻿﻿South Australian Museum (**SAMA**; Adelaide, South Australia, Australia), ﻿South African Museum (**SAMC**; Iziko Museums of South Africa, Cape Town, South Africa), and Utah State University Insect Collection (**USU**; Logan, Utah).

Each specimen examined in this paper has a unique identifier consisting of a prefix (e.g., “OSUC”) and a number. The associated data for each specimen may be accessed at http://mbd-db.osu.edu using this unique identifier. Morphological terminology generally follows Mikó et al. (2007). The term claval formula (Bin 1981) refers to the apical antennomeres of the female that bear papillary sensilla on their ventral surface. The claval formula is the number of papillary sensilla on each antennomere separated by a dash, starting from the distal antennomere to the most proximal antennomere. The antennomere is also designated by number (from proximal to distal segment). A 5–segmented clava with 1 sensillum on the most distal antennomere and 2 sensilla on each of the remaining antennomeres would be represented with a claval formula of A12–A8: 1–2–2–2–2. Metasomal tergites are referred to by the letter T followed by a number, e.g., T1 is the first (i.e., basalmost) metasomal tergite.

The terminology for the surface sculpture follows (Harris, 1979). Species descriptions and a taxon by data matrix were generated using vSysLab (https://vsyslab.osu.edu). These descriptions were exported in the format of “Character: Character state(s).” The states of characters polymorphic for a species are separated by semicolons. Photographs of specimens were captured using a Leica Z16 APOA system and stacked with the Leica Application Suite software. Images of type specimens were provided by Elijah Talamas (Florida State Collection of Arthropods).

Our concept of species is based on the biological species concept as described by Mayr (1942). Species are populations whose individuals have the ability to interbreed in nature. Many morphological characters likely are the result of polygenic origin, and interbreeding populations exchange genes among themselves but not with other species. Thus, one would predict that separate species will eventually come to evolve differences in morphological character states, either randomly or through natural selection (Wild 2004). Other factors – including sexual dimorphism, under-sampling of intraspecific variability, genetically simple but discrete character states, and environmental influences – may suggest species differences. It is the task of the taxonomist in the early stages of the study of a group to tease apart such sources of variation, evaluate the evidence, and propose hypotheses of how many independent species exist and which characters can be used to distinguish them. Going forward, these hypotheses can be tested with new characters and new sources of characters.

## ﻿Results

### ﻿Key to species of *Dichoteleas*

**Table:** 

1	Interantennal process produced anterodorsally, pinched laterally, surrounded by depression, central keel on frons present (Fig. 22); India	***Dichoteleasindicus***
–	Interantennal process flattened against lower frons; central keel absent (Fig, 10)	**2**
2	Median carina on T1–T4 present (Fig. 14), head metallic blue	**3**
–	Median carina on T1–T4 absent, head black (Fig. 21)	**5**
3	Median mesoscutal line present (Fig. 37), axillular carinae xanthic; Australia (Queensland)	***Dichoteleassubcoeruleus***
–	Median mesoscutal line absent (Figs 11, 15), axillular carinae variable, concolorous with mesosoma or only slightly lighter	**4**
4	Mesoscutum finely punctate (Fig. 15), pronotum metallic blue (Fig. 12), submedian carinae absent on frons (Fig. 13); Indonesia (West Papua)	***Dichoteleasfulgidus* sp. nov.**
–	Mesoscutum rugulose (Fig. 18), pronotum black to dark brown (Fig. 16); submedian carinae present on frons (Fig. 17); Papua New Guinea, Australia (Queensland)	***Dichoteleasfuscus* sp. nov.**
5	Notaulus incomplete, pronotum xanthic (Fig. 11); Madagascar	***Dichoteleasambositrae***
–	Notaulus complete, pronotum red, black, or brown (Fig. 28, 31, 34)	**6**
6	Axillular carinae with a laterally compressed, posteroventral hooklike projection (Fig. 19); South Africa (Limpopo), Malawi, Kenya, Tanzania	***Dichoteleashamatus* sp. nov.**
–	Axillular carinae triangular and pointed posteriorly or slightly curved inwardly (Fig. 28)	**7**
7	Mesosoma red dorsally and darkened posteroventrally, areolate-rugose (Figs 26, 28); Madagascar	***Dichoteleasrubyae* sp. nov.**
–	Mesosoma dark brown to black, punctate or with longitudinal striations between notauli beginning posteriorly (Fig. 31, 40)	**8**
8	Mesoscutum and scutellum smooth with sparse setation (Fig. 34); Madagascar	***Dichoteleasstriatus* sp. nov.**
–	Mesoscutum and scutellum setose and punctate (Fig. 31)	**9**
9	Mandibles bidentate; mesoscutual humeral sulcus foveolate; mesoscutum with longitudinal striations between notauli beginning posteriorly (Figs 29, 30); Australia (Queensland)	***Dichoteleasrugosus***
–	Mandibles tridentate; mesoscutual humeral sulcus present as an uninterrupted groove; mesoscutum punctate between notauli with xanthic posterolateral corners (Figs 39, 40); Tanzania	***Dichoteleasumbra* sp. nov.**

#### ﻿ Dichoteleas

Taxon classificationAnimaliaHymenopteraScelionidae

Kieffer

BE466549-D214-5E96-9B07-0466937F17B1

Dichoteleas Kieffer: 1907: 297: (original description. Type: Dichoteleasrugosus Kieffer, by monotypy); Brues: 1908: 28, 44: (diagnosis, list of species, keyed); Kieffer: 1908: 113: (keyed); Kieffer: 1910: 62: (keyed); Dodd: 1913: 131: (keyed); Kieffer: 1913: 23: (description); Dodd: 1926: 369: (description, key to species); Kieffer: 1926: 266, 351: (description, keyed); Muesebeck & Walkley: 1956: 346: (citation of type species); Masner: 1976: 30: (description); Mani & Sharma: 1982: 173: (description); Galloway & Austin: 1984: 7, 16: (diagnosis, list of species described from Australia, keyed); Johnson: 1992: 367: (cataloged, catalog of world species); Rajmohana: 2006: 116, 123: (description, keyed).

##### Description.

***Head*.** Head shape in dorsal view: transverse. Vertex: smooth or rugose. Hyperoccipital carina: present or absent. Occipital carina: present, complete. OOL: lateral ocellus nearly contiguous with inner orbits, OOL < 0.5 OD. Upper frons: convex or with a slight concavity; smooth, striate, or areolate. Frontal depression: undifferentiated. Submedian carina: present or absent. Orbital carina: present. Inner orbits: diverging ventrally. IOS/EH: IOS less than EH. Interantennal process: short, often excavate medially. Central keel: present or absent. Antennal foramen: oriented laterally on interantennal process. Facial striae: present or absent. Malar sulcus: present. Malar striae: present or absent. Setation of compound eye: present or absent. Gena: narrows dorsally behind eye, convex. Clypeus shape: narrow, rectangular, lateral corners not produced. Anterior (or ventral) margin of clypeus: straight. Labrum: narrow, trapezoidal, ventral margin convex or straight. Number of mandibular teeth: 2 or 3. Arrangement of mandibular teeth: transverse. Number of maxillary palpomeres: 4. Shape of maxillary palpomeres: cylindrical. Number of labial palpomeres: 2.

***Antenna*.** Number of antennomeres in female: 12. Number of antennomeres in male: 12. Insertion of radicle into A1: parallel to longitudinal axis of A1. Shape of A1: cylindrical, not flattened. Length of A3 of female: distinctly longer than A2. Number of antennomeres with papillary sensilla in female: 7. Arrangement of sensilla on female clava: in longitudinal pairs. Claval formula: A12–A6:1–2–2–2–2–2–2. Shape of male flagellum: filiform. Sex segment of male antenna: A5.

***Mesosoma*.** Posterior apex of pronotum in dorsal view: bifid apically to articulate with tegula. Epomial carina: absent. Cervical pronotal area: oblique, visible dorsally, short. Lateral face of pronotum: weakly concave ventrally around the pronotal cervical sulcus. Netrion: present. Netrion shape: moderately wide, open ventrally. Anterior portion of mesoscutum: vertical, flexed ventrally to meet pronotum. Mesoscutum shape: pentagonal, excavate at base of wings. Skaphion: absent. Notauli: present, percurrent. Parapsidal lines: present. Antero-admedian lines: absent. Transscutal articulation: well-developed. Mesoscutal suprahumeral sulcus: present or absent. Mesoscutal humeral sulcus: present as an uninterrupted groove or foveolate. Shape of mesoscutellum: trapezoidal. Lateral mesoscutellar spines: present. Median mesoscutellar spine: absent. Axillular spines: present. Surface of mesoscutellum: convex throughout. Median longitudinal furrow on mesoscutellum: absent. Metascutellum: clearly differentiated. Shape of metascutellum: flattened laterally into a medially spine; flattened dorsoventrally into a triangular plate. Setation of metascutellum: absent. Metapostnotum: fused to propodeum. Lateral propodeal projection: absent. Medial propodeal projection: absent. Mesopleural carina: present. Mesal course of acetabular carina: not separating fore coxae. Mesopleural pit: present. Posterodorsal corner of mesopleuron: rounded.

***Legs*.** Number of mesotibial spurs: 1. Number of metatibial spurs: 1. Dorsal surface of metacoxa: smooth. Shape of metacoxa: cylindrical, ecarinate. Trochantellus: indicated by transverse sulcus on femur.

***Wings*.** Wing development of female: macropterous. Wing development of male: macropterous. Tubular veins in fore wing: present. Bulla of fore wing R: absent. Length of marginal vein of fore wing: punctiform, R terminating at costal margin. Origin of r-rs in fore wing: arises at the point where R meets costal margin. Development of R in hind wing: complete.

***Metasoma*.** Number of external metasomal tergites in female: 7. Number of external metasomal sternites in female: 7. Number of external metasomal tergites in male: 8. Number of external metasomal sternites in male: 7. Shape of metasoma: lanceolate. Laterotergites: present, narrow. Laterosternites: present. T1 of female: flat; produced anteriorly as a small hump. Relative size of metasomal segments: T2–T3 subequal in length, remaining terga shorter. Metasomal tergites with basal crenulae: T2. Sublateral carinae on tergites: present. Median longitudinal carina on metasomal terga: absent; present on T1–T4. Shape of female T6: slightly convex. Anterior margin of S1: not produced anteriorly, straight. Felt fields on S2: present; obscured by setation. Felt fields on S3: present; obscured by setation. Ovipositor: *Scelio*-type (Austin and Field 1997).

##### Generic diagnosis.

*Dichoteleas* can be identified by its elongate maxillary palpi, lateral spines on the mesoscutellum, medial spine on the metascutellum, and well-developed postmarginal vein on the forewing. This taxon can be distinguished from *Neoscelio* by the short (or absent) setation on the eyes and the well-developed postmarginal vein. It may be distinguished from *Oxyteleia* and *Oreiscelio* since in *Dichoteleas* the metascutellum only has a single median spine. The New World genus *Pseudoheptascelio* may also be interpreted to have a bidentate mesoscutellum. In that group the stigmal vein (r-rs) arises from the submarginal vein before it reaches the costal margin of the fore wing. In *Dichoteleas*, the stigma vein arises from the costal margin.

##### Distribution.

*Dichoteleas* species are known from Kenya, Tanzania, Malawi, northeastern South Africa, Madagascar, southern India, New Guinea and Far North Queensland in Australia (Figs 1–8). No specimens have yet been collected in other parts of sub-Saharan Africa, southeast Asia, or regions to the east of Papua New Guinea.

**Figures 1–8.**
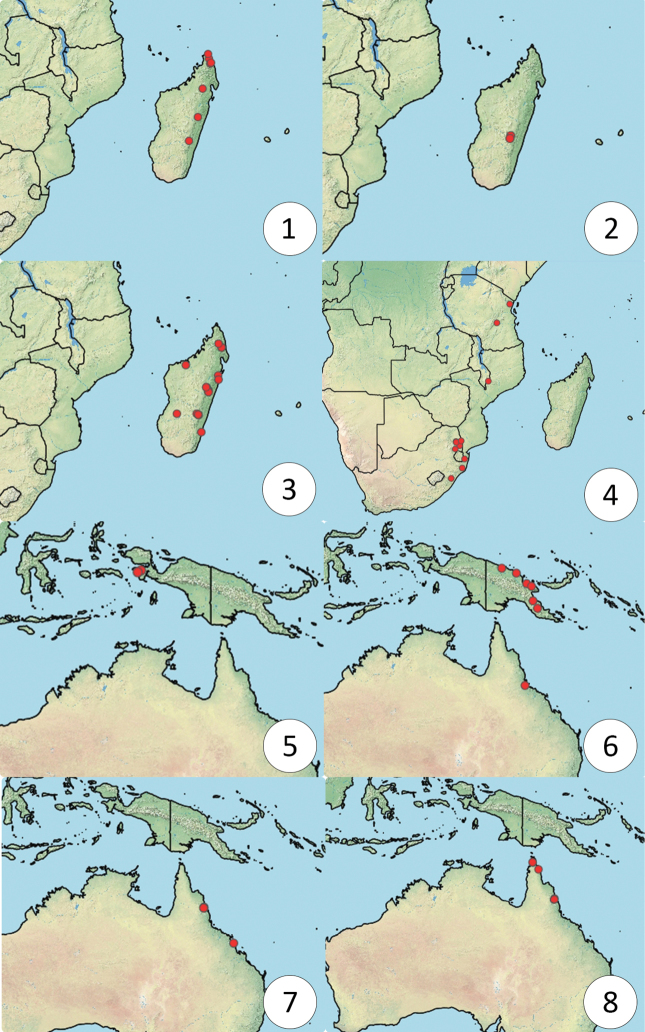
Distribution of *Dichoteleas* species **1***D.rubyae***2***D.ambositrae***3***D.striatus***4***D.hamatus***5***D.fulgidus***6***D.fuscus***7***D.rugosus***8***D.subcoeruleus*.

#### ﻿ Dichoteleas ambositrae

Taxon classificationAnimaliaHymenopteraScelionidae

Risbec

B346EC6A-90A9-5869-BA0A-D98120E3544D

Figs 2 , 9–11

Dichoteleia ambositrae Risbec, 1956: 261 (original description).
Dichoteleas ambositrae : Masner, 1976: 31 (type information); Johnson, 1992: 367 (catalogued, type information).

##### Description.

Color of head: black. Hyperoccipital carina: present. Frontal depression: absent. Malar striae: absent. Facial striae: present. Setation of eyes: absent. Sculpture of frons: primarily smooth with weak transverse striations above the IAP. Setation of frons: mostly glabrous with sparse setation laterally. Submedian carina: absent. Interantennal process: undifferentiated. Central keel: absent. Transverse pronotal carina: absent. Color of pronotum: yellow. Pronotal cervical sulcus: present. Mesepimeral sulcus: present. Sulcus along mesopleural carina: foveolate. Mesoscutal suprahumeral sulcus: present. Mesoscutal humeral sulcus: present as an uninterrupted groove. Median mesoscutal line: absent. Color of mesoscutum: dark brown to black. Sculpture of mesoscutum: smooth without longitudinal striations. Notaulus: incomplete. Visibility of notaulus: unobscured. Parapsidal line: present. Sculpture of mesoscutellum: smooth. Shape of axillular carinae in lateral view: without a posteroventral hooklike projection. Color of axillular carina: yellow. Sculpture of T3–6: punctate. Median carina on T1–T4: absent.

##### Diagnosis.

This species can be distinguished from *D.subcoeruleus*, *D.fulgidus*, *and D.fuscus* by the absence of the median carina on T1–T4. It can be distinguished from the other species by its xanthic pronotum and smooth mesoscutum.

##### Distribution.

Madagascar (Ambositra, Fianarantsoa).

##### Material examined.

***Holotype***, female: **Madagascar**: Ambositra, MNHN Paris EY32526; **Madagascar**: 2 females, CASENT 2138155, 2138157 (CAS).

#### ﻿ Dichoteleas fulgidus sp. nov.

Taxon classificationAnimaliaHymenopteraScelionidae

6090B03D-9849-5263-8E7A-E783BD2FCBDA

https://zoobank.org/551D6CCD-7E66-496A-9B7C-53D007349DAC

Figs 5 , 12–15

##### Description.

Color of head: metallic blue. Hyperoccipital carina: present. Frontal depression: absent. Malar striae: absent. Facial striae: absent. Setation of eyes: absent. Sculpture of frons: smooth above interantennal prominence, areolate laterally. Setation of frons: sparsely setose throughout. Submedian carina: absent. Interantennal process: undifferentiated. Central keel: absent. Transverse pronotal carina: present. Color of pronotum: metallic blue. Pronotal cervical sulcus: absent. Mesepimeral sulcus: absent. Sulcus along mesopleural carina: absent. Mesoscutal suprahumeral sulcus: absent. Mesoscutal humeral sulcus: present as an uninterrupted groove. Median mesoscutal line: absent. Color of mesoscutum: metallic blue. Sculpture of mesoscutum: finely punctate without longitudinal striations. Notaulus: complete. Visibility of notaulus: unobscured. Parapsidal line: present. Sculpture of mesoscutellum: smooth. Shape of axillular carinae in lateral view: without a posteroventral hooklike projection. Color of axillular carina: metallic blue. Sculpture of T3–6: rugulose. Median carina on T1–T4: present.

**Figures 9–11.**
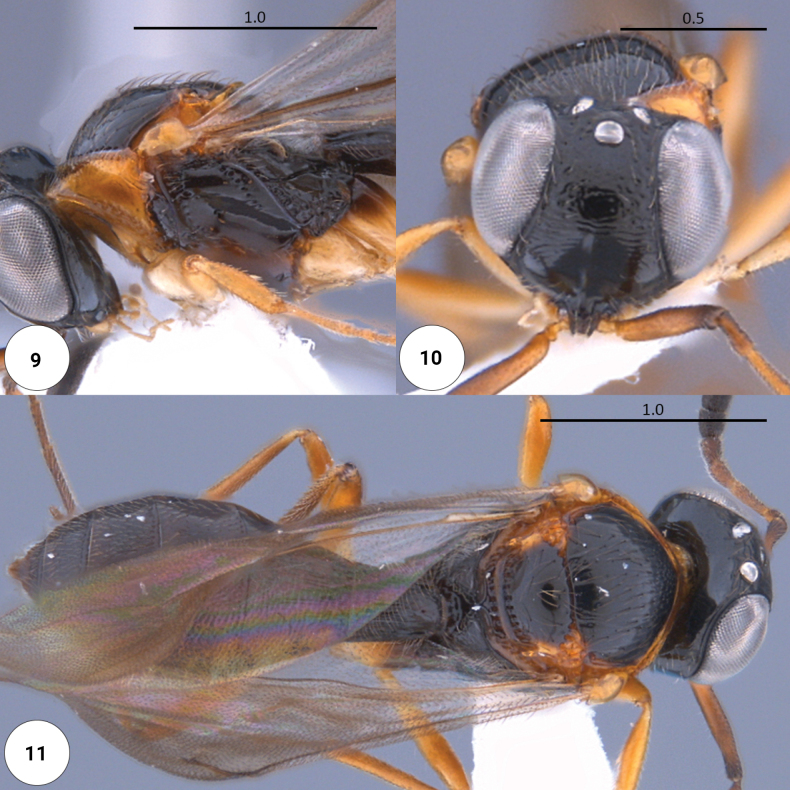
*Dichoteleasambositrae* (F) (CASENT 2138155) **9** head, mesosoma, lateral view **10** head, anteroventral view **11** dorsal habitus.

##### Diagnosis.

This species can be identified by the presence of a dorsal median carina on T1–T4 of the metasoma, and it may be distinguished from *D.subcoeruleus* and *D.fuscus* by the finely punctate sculpture of the mesoscutum.

##### Etymology.

The epithet comes from the Latin word for “shiny,” referring to the smooth, metallic luster of the mesosoma. This epithet is treated as an adjective.

##### Distribution.

Indonesia (Papua Barat).

##### Material examined.

***Holotype***, female: **Indonesia**: FakFak S. coast of Bomberai, 100–700m; OSUC 234427 (BPBM). *Paratypes*. **Indonesia**: 3 males, OSUC 234420–234421, 234425 (BPBM).

**Figures 12–15.**
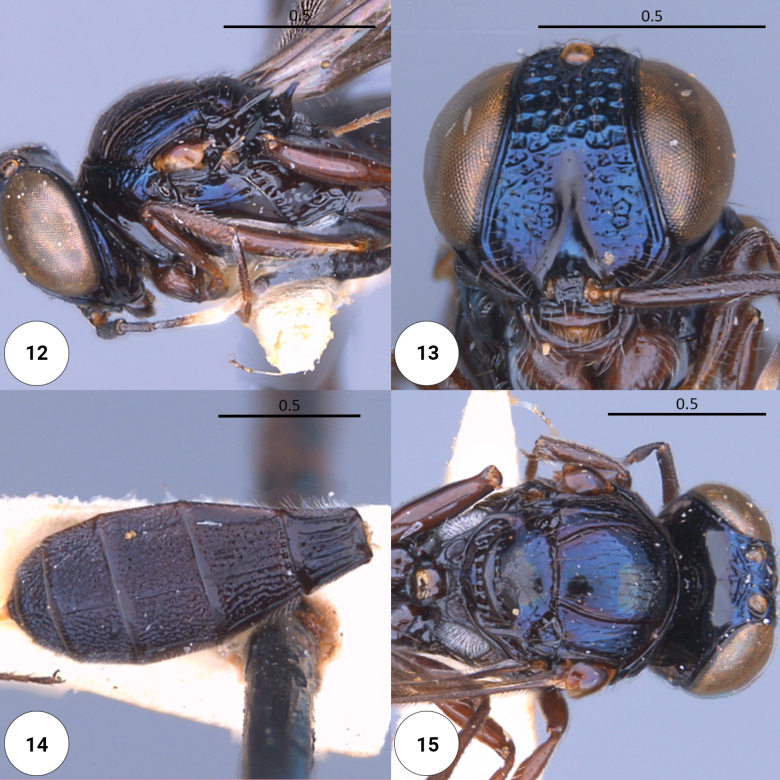
*Dichoteleasfulgidus* (F) (OSUC 0234427) **12** head, mesosoma, lateral view **13** head, anteroventral view **14** metasoma, dorsal view **15** head, mesosoma, dorsal view.

#### ﻿ Dichoteleas fuscus sp. nov.

Taxon classificationAnimaliaHymenopteraScelionidae

CF71C170-0479-5758-ADFB-450377D84DFD

https://zoobank.org/8CA7BC20-6A81-4DBE-904A-2997CA6072F3

Figs 6 , 16–18

##### Description.

Color of head: metallic blue. Hyperoccipital carina: present. Frontal depression: absent. Malar striae: absent. Facial striae: absent. Setation of eyes: absent. Sculpture of frons: smooth above interantennal prominence, areolate laterally. Setation of frons: sparsely setose throughout. Submedian carina: present. Interantennal process: undifferentiated. Central keel: absent. Transverse pronotal carina: present. Color of pronotum: dark brown to black. Pronotal cervical sulcus: absent. Mesepimeral sulcus: absent. Sulcus along mesopleural carina: absent. Mesoscutal suprahumeral sulcus: absent. Mesoscutal humeral sulcus: present as an uninterrupted groove. Median mesoscutal line: absent. Color of mesoscutum: dark brown to black. Sculpture of mesoscutum: rugulose. Notaulus: complete. Visibility of notaulus: unobscured. Parapsidal line: present. Sculpture of mesoscutellum: rugulose. Shape of axillular carinae: without a posteroventral hooklike projection. Color of axillular carina: brown. Sculpture of T3–6: rugulose. Median carina on T1–T4: present.

**Figures 16–18.**
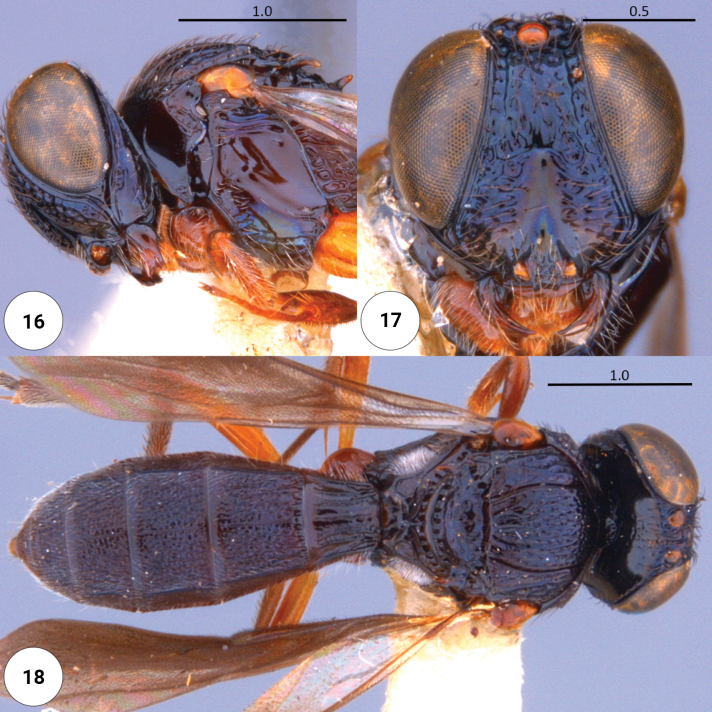
*Dichoteleasfuscus* (F) (OSUC 0234418) **16** head, mesosoma, lateral view **17** head, anteroventral view **18** dorsal habitus.

##### Diagnosis.

This species can be identified by the dorsal median carina (T1–T4 of metasoma) and can be distinguished from *D.subcoeruleus* and *D.fulgidus* by the rugulose sculpture of the mesoscutum.

##### Etymology.

The epithet comes from the Latin word for “dusky,” referring to the darker, metallic color of the mesosoma (in comparison to *D.fulgidus*). This epithet is treated as an adjective.

##### Distribution.

Papua New Guinea (Madang, Morobe, Northern, East Sepik), Australia (Queensland).

##### Material examined.

***Holotype***, female: **Papua New Guinea**: NE Finisterre Range, Saidor, Gabumi; OSUC 234417 (BPBM). ***Paratypes*. Australia**: 1 male, OSUC 875045 (CNCI). **Papua New Guinea**: 12 females, 4 males, OSUC 234413–234416, 234422–234424, 234426, 234428–234429 (BPBM), OSUC 875873–875876 (CNCI).

#### ﻿ Dichoteleas hamatus sp. nov.

Taxon classificationAnimaliaHymenopteraScelionidae

8EE29820-64E5-54A0-9841-703FD18378F7

https://zoobank.org/09D49988-1679-4AC2-AC59-D5C47ED6E353

Figs 4 , 19–21

##### Description.

Color of head: black. Hyperoccipital carina: absent. Frontal depression: absent. Malar striae: present. Facial striae: present. Setation of eyes: sparse, with few scattered fine hairs. Sculpture of frons: smooth above interantennal prominence, areolate laterally. Setation of frons: sparsely setose throughout. Submedian carina: absent. Interantennal process: undifferentiated. Central keel: absent. Transverse pronotal carina: present. Color of pronotum: dark brown to black. Pronotal cervical sulcus: present. Mesepimeral sulcus: present. Sulcus along mesopleural carina: absent. Mesoscutal suprahumeral sulcus: absent. Mesoscutal humeral sulcus: present, foveolate. Median mesoscutal line: absent. Color of mesoscutum: dark brown to black. Sculpture of mesoscutum: areolate-rugose. Notaulus: complete. Visibility of notaulus: slightly obscured by mesoscutal sculpture. Parapsidal line: present. Sculpture of mesoscutellum: rugulose. Shape of axillular carinae in lateral view: with a sharp posteroventral hooklike projection. Color of axillular carina: brown. Sculpture of T3–6: rugulose and finely punctate. Median carina on T1–T4: absent.

##### Diagnosis.

This species can be distinguished from *D.rugosus* by the distinct hooked projections on axillular carinae.

##### Etymology.

The name *hamatus* is drawn from the Latin word for hooked, referring to the hooked projections on the axillular carinae. This epithet is treated as an adjective.

##### Distribution.

Kenya (Coast), Malawi (Mulanje), South Africa (Limpopo), Tanzania (Uzungwa Mts., Tanga Amani Hills).

##### Material examined.

***Holotype***, female: **South Africa**: Guernsey Farm, Limpopo Prov.; OSUC 56306 (CNCI). *Paratypes*. **Kenya**: 1 female, ICIPE 32195 (ICIPE). **Malawi**: 1 female, OSUC 875032 (CNCI). **South Africa**: 20 females, 50 males, OSUC 874965–875031, 875037 (CNCI); SAM-HYM-P031302, SAM-HYM-P037851 (SAMC); USNMENT01197871 (USNM). **Tanzania**: 5 females, 1 male, OSUC 875033–875036, 875040-875041 (CNCI).

#### ﻿ Dichoteleas indicus

Taxon classificationAnimaliaHymenopteraScelionidae

Saraswat

AAEAF8D2-BA5C-5D80-B539-C6F21052D530

Figs 22–25

Dichoteleas indicus Saraswat, 1982: 350 (original description); Johnson, 1992: 367 (catalogued, type information).

##### Description.

Color of head: black. Hyperoccipital carina: present. Frontal depression: absent. Malar striae: present. Facial striae: present. Setation of eyes: sparse, with few scattered fine hairs. Sculpture of frons: primarily rugulose. Setation of frons: sparsely setose throughout. Submedian carina: present. Interantennal process: produced anteriorly, margined by depression. Central keel: present. Transverse pronotal carina: present. Color of pronotum: dark brown to black. Pronotal cervical sulcus: present. Mesepimeral sulcus: absent. Sulcus along mesopleural carina: absent. Mesoscutal suprahumeral sulcus: absent. Mesoscutal humeral sulcus: present as an uninterrupted groove. Median mesoscutal line: present. Color of mesoscutum: dark brown to black. Sculpture of mesoscutum: rugulose. Notaulus: complete. Visibility of notaulus: slightly obscured by mesoscutal sculpture. Parapsidal line: present. Sculpture of mesoscutellum: rugulose. Shape of axillular carinae in lateral view: without a posteroventral hooklike projection. Color of axillular carina: yellow. Sculpture of T3–6: strigate and finely punctate. Median carina on T1–T4: absent.

**Figures 19–21.**
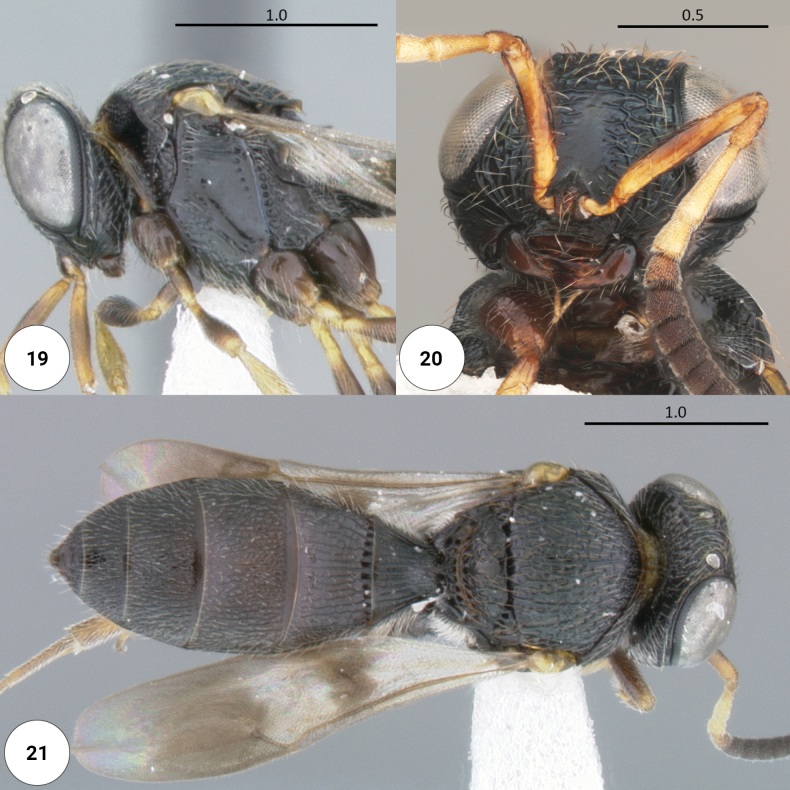
*Dichoteleashamatus* (F) (OSUC 56306) **19** head, mesosoma, lateral view **20** head, anteroventral view **21** dorsal habitus.

**Figures 22–25.**
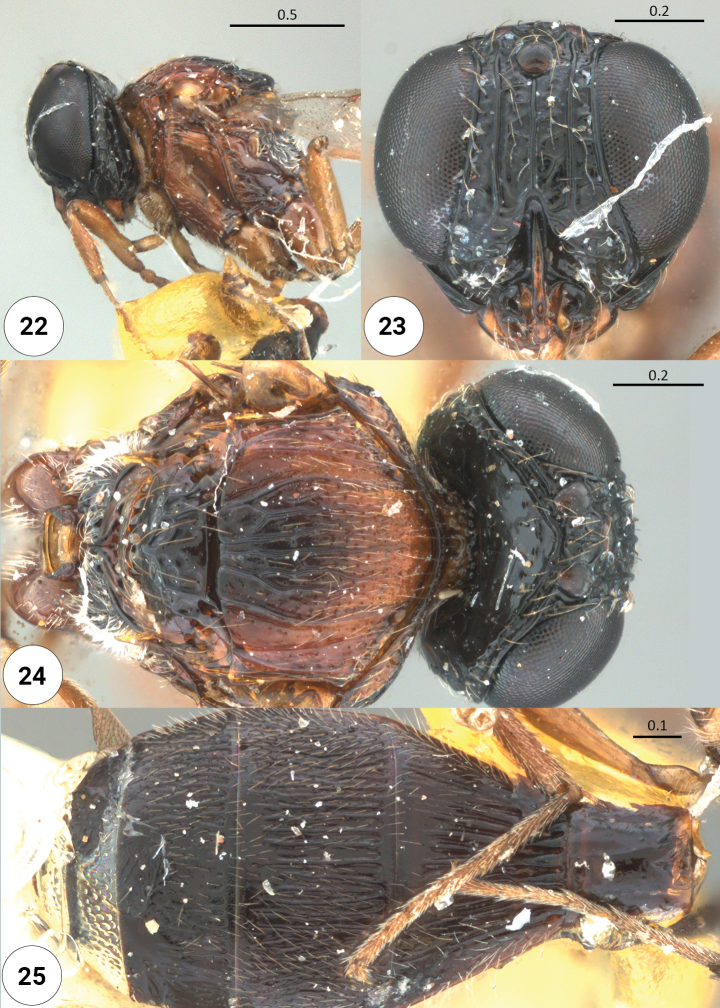
*Dichoteleasindicus* (M) (USNMENT 01109962) **22** mesosoma, lateral view **23** head, anteroventral view **24** head, mesosoma, dorsal view **25** metasoma, dorsal view.

##### Diagnosis.

This species can be distinguished by the anteriorly produced interantennal process and the presence of the central keel.

##### Distribution.

India (Kerala).

##### Material examined.

Holotype, male: **India**: School of Entomology, St. John’s College; USNMENT 01109962. **India**: 1 female, OSUC 875044 (CNCI).

#### ﻿ Dichoteleas rubyae sp. nov.

Taxon classificationAnimaliaHymenopteraScelionidae

683C18AF-8826-5B08-8C8B-D9C0307CD016

https://zoobank.org/A33B1DD4-261B-4969-957E-26E7C3D654A1

Figs 1 , 26–28

##### Description.

Color of head: black. Hyperoccipital carina: absent. Frontal depression: present, shallow. Malar striae: present. Facial striae: present. Setation of eyes: absent. Sculpture of frons: smooth above interantennal prominence, areolate laterally. Setation of frons: sparsely setose throughout. Submedian carina: absent. Interantennal process: undifferentiated. Central keel: absent. Transverse pronotal carina: absent. Color of pronotum: red. Pronotal cervical sulcus: present. Mesepimeral sulcus: present. Sulcus along mesopleural carina: foveolate. Mesoscutal suprahumeral sulcus: present. Mesoscutal humeral sulcus: present, foveolate. Median mesoscutal line: absent. Color of mesoscutum: red. Sculpture of mesoscutum: areolate-rugose. Notaulus: complete. Visibility of notaulus: slightly obscured by mesoscutal sculpture. Parapsidal line: present. Sculpture of mesoscutellum: smooth. Shape of axillular carinae: without a posteroventral hooklike projection. Color of axillular carina: yellow. Sculpture of T3–6: rugulose and finely punctate. Median carina on T1– T4: absent.

##### Diagnosis.

This species can be distinguished from *D.rugosus* by its reddish mesosoma and the smooth mesoscutellum.

##### Etymology.

The epithet *rubyae* in honor of the first author’s grandmother, Ruby Thomas. The name also refers to the red coloration of the mesosoma. This epithet is treated as a noun in the genitive case.

##### Distribution.

Madagascar (Antsiranana, Ranomafana).

##### Material examined.

***Holotype***, female: **Madagascar**: Prov. Antsiranana, Forêt de Binara, 375m; CASENT 2134207 (CAS). ***Paratypes*. Madagascar**: 19 females, 7 males, CASENT 2043443–2043447, 2131300–2131302, 2134208–2134212, 2137245, 2137863 (CAS), OSUC 874887, 874942 (CNCI); CASENT 2042724–2042725, 2043436-2043442, 2134212, 2137245, 2137863 (OSUC).

##### Comments.

There is some variation in the visibility of the notauli. In most specimens, the notauli were obscured by the mesoscutal sculpture, but one specimen (CASENT 2137863) had clearly defined notauli.

#### ﻿ Dichoteleas rugosus

Taxon classificationAnimaliaHymenopteraScelionidae

Kieffer

BED2E917-4A7B-5C93-BE08-F7E2A849A957

Figs 7 , 29–31

Dichoteleas rugosus Kieffer, 1907: 297 (original description); Kieffer, 1926: 351 (description, keyed); Dodd, 1926: 370 (description); Masner, 1965: 72 (type information); Galloway, 1976: 90 (type information); Johnson, 1992: 367 (catalogued, type information).
Dichoteleas pappi Szabó, 1971: 319 (original description); Galloway 1976: 90 (type information); Johnson, 1992: 367 (catalogued, type information), new synonymy.

##### Description.

Color of head: black. Hyperoccipital carina: absent. Frontal depression: absent. Malar striae: present. Facial striae: present. Setation of eyes: absent. Sculpture of frons: smooth above interantennal prominence, areolate laterally. Setation of frons: sparsely setose throughout. Submedian carina: absent. Interantennal process: undifferentiated. Central keel: absent. Transverse pronotal carina: present. Color of pronotum: dark brown to black. Pronotal cervical sulcus: present. Mesepimeral sulcus: present. Sulcus along mesopleural carina: foveolate. Mesoscutal suprahumeral sulcus: absent. Mesoscutal humeral sulcus: present, foveolate. Median mesoscutal line: absent. Color of mesoscutum: dark brown to black. Sculpture of mesoscutum: punctate with longitudinal striations between notauli. Notaulus: complete. Visibility of notaulus: unobscured. Parapsidal line: present. Sculpture of mesoscutellum: punctate. Shape of axillular carinae in lateral view: without a posteroventral hooklike projection. Color of axillular carina: brown. Sculpture of T3–6: rugulose and finely punctate. Median carina on T1–T4: absent.

**Figures 26–28.**
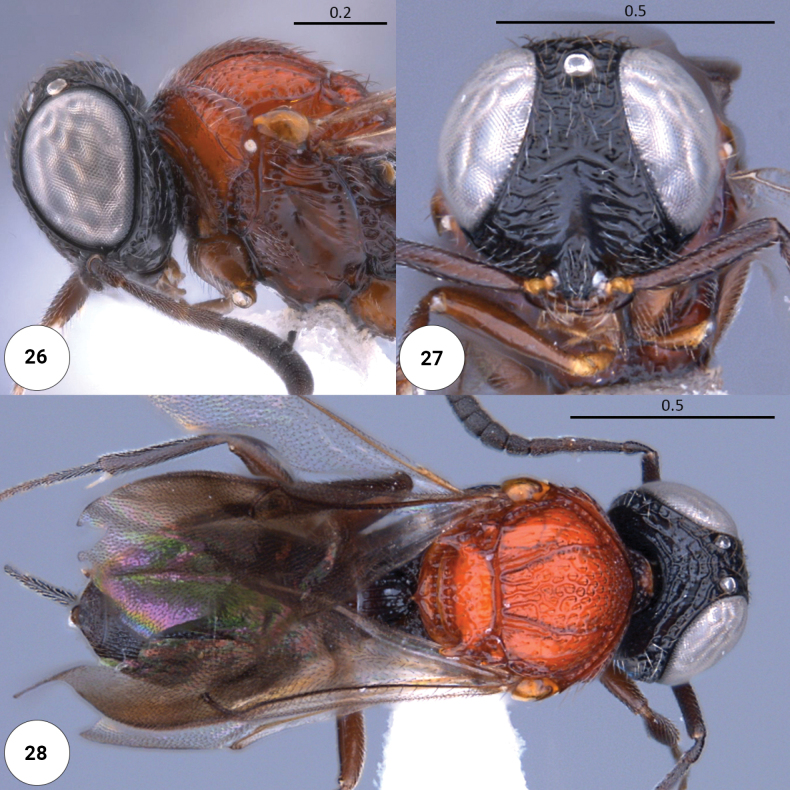
*Dichoteleasrubyae* (F) (CASENT 2137863) **26** head, mesosoma, lateral view **27** head, anteroventral view **28** dorsal habitus.

**Figures 29–31.**
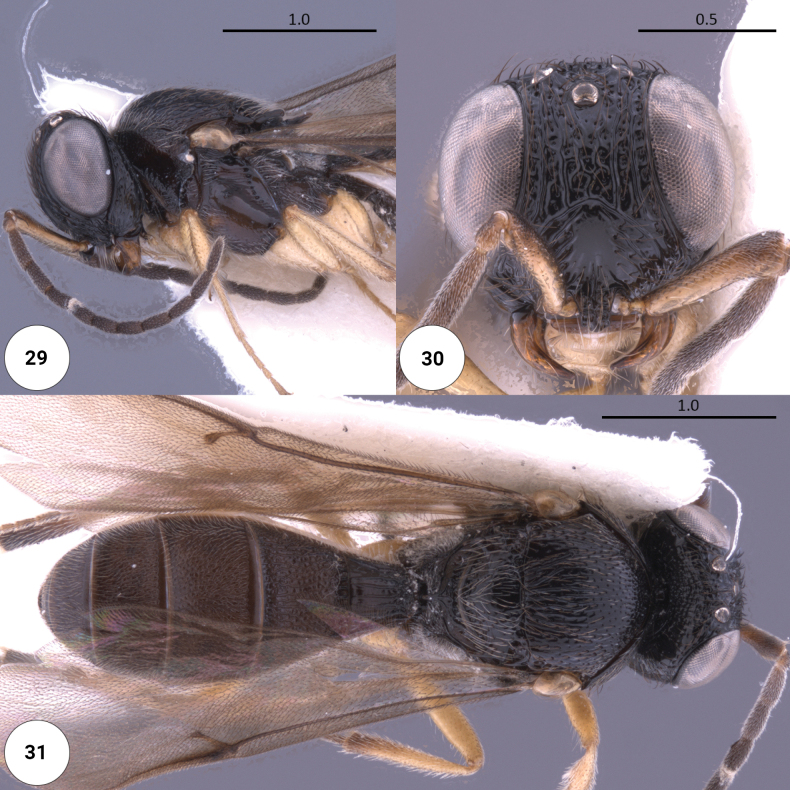
*Dichoteleasrugosus* (M) (OSUC 0367523) **29** head, mesosoma, lateral view **30** head, anteroventral view **31** dorsal habitus.

##### Diagnosis.

*Dichoteleasrugosus* can be distinguished from *D.striatus* by its setose and punctate mesosoma and other *Dichoteleas* by its bidentate mandibles.

##### Distribution.

Australia (Queensland).

##### Material examined.

***Holotype***, male, *D.rugosus*: **Australia**: QLD, Mackay; OCT-1897, B.M. TYPE HYM. 9.496.; **Australia**: 4 females, 2 males, OSUC 367523, 367536 (ANIC), OSUC 875046–875047, 875871–875872 (CNCI).

##### Comments.

In the original description, Kieffer (1907) wrote that *D.rugosus* was missing parapsidal lines. They are present but obscured by the sculpture of the mesoscutum.

#### ﻿ Dichoteleas striatus sp. nov.

Taxon classificationAnimaliaHymenopteraScelionidae

CE5BC783-73CD-5F07-9DBD-D2F5EB4EC053

https://zoobank.org/985F5CD2-7A6E-4546-8CDA-FCFF2BBFE104

Figs 3 , 32–34

##### Description.

Color of head: black. Hyperoccipital carina: present. Frontal depression: absent. Malar striae: absent. Facial striae: present. Setation of eyes: absent. Sculpture of frons: primarily smooth with weak transverse striations above the IAP. Setation of frons: mostly glabrous with sparse setation laterally. Submedian carina: absent. Interantennal process: undifferentiated. Central keel: absent. Transverse pronotal carina: present. Color of pronotum: dark brown to black. Pronotal cervical sulcus: present. Mesepimeral sulcus: present. Sulcus along mesopleural carina: absent. Mesoscutal suprahumeral sulcus: present. Mesoscutal humeral sulcus: present as an uninterrupted groove. Median mesoscutal line: absent. Color of mesoscutum: dark brown; black. Sculpture of mesoscutum: primarily smooth with longitudinal striations between notauli. Notaulus: complete. Visibility of notaulus: slightly obscured by mesoscutal sculpture. Parapsidal line: present. Sculpture of mesoscutellum: smooth. Shape of axillular carinae in lateral view: without a posteroventral hooklike projection. Color of axillular carina: brown. Sculpture of T3–6: weakly strigate and finely punctate. Median carina on T1–T4: absent.

##### Diagnosis.

This species can be distinguished from *D.ambositrae* by the longitudinal striations between the notauli and the black/brown pronotum and from *D.rugosus* by its glabrous mesosoma.

##### Etymology.

The epithet refers to the longitudinal striations present on the mesoscutum. This epithet is treated as an adjective.

##### Distribution.

Madagascar (Antananarivo, Antsiranana, Fianarantsoa, Mahajanga, Toamasina).

##### Material examined.

***Holotype***, female: **Madagascar**: Prov. Fianarantsoa, 1130m, PN Ranomafana, radio tower; CASENT 2043988 (CAS). ***Paratypes*. Madagascar**: 93 females, 64 males, CASENT 2043198, 2043540, 2043564–2043565, 2043989, 2118400, 2118404, 2118444, 2131303–2131316, 2132729, 2132737, 2133921–2133922, 2134086, 2134150, 2134156, 2134161, 2134169, 2134198–2134200, 2134203– 2134204, 2134523, 2135869, 2135989, 2136263, 2136415, 2137236, 2137832, 2137876, 2137937, 2138216 (CAS); CASENT 2042824, 2042844–2042857, 2042967–2042976, OSUC 146657, 229802 (OSUC); OSUC 218026 (USU); OSUC 874879–874883, 874885–874886, 874888–874941, 874943–874964 (CNCI).

##### Comments.

There was some variation in the length and the number of the longitudinal striations on the mesoscutum. In fewer than half of the specimens, the striations started anteriorly and terminated around the middle of the mesoscutum. In the majority of the specimens, the striations started anteriorly and terminated at the posterior margin of the mesoscutum.

#### ﻿ Dichoteleas subcoeruleus

Taxon classificationAnimaliaHymenopteraScelionidae

Dodd

82147B10-F745-5257-B6F7-FE7446A2F4B3

Figs 8 , 35–37

Dichoteleas subcoeruleus Dodd, 1926: 370, 371 (original description); Galloway 1976: 90 (type information); Johnson, 1992: 367 (catalogued, type information).

##### Description.

Color of head: metallic blue. Hyperoccipital carina: absent. Frontal depression: absent. Malar striae: absent. Facial striae: present. Setation of eyes: absent. Sculpture of frons: smooth above interantennal prominence, areolate laterally. Setation of frons: sparsely setose throughout. Submedian carina: present. Interantennal process: undifferentiated. Central keel: absent. Transverse pronotal carina: present. Color of pronotum: dark brown to black. Pronotal cervical sulcus: absent. Mesepimeral sulcus: present. Sulcus along mesopleural carina: foveolate. Mesoscutal suprahumeral sulcus: absent. Mesoscutal humeral sulcus: present as an uninterrupted groove. Median mesoscutal line: present. Color of mesoscutum: metallic blue. Sculpture of mesoscutum: rugulose. Notaulus: complete. Visibility of notaulus: unobscured. Parapsidal line: present. Sculpture of mesoscutellum: smooth. Shape of axillular carinae in lateral view: without a posteroventral hooklike projection. Color of axillular carina: yellow. Sculpture of T3–4: rugulose and finely punctate. Median carina on T1–T4: present.

**Figures 32–34.**
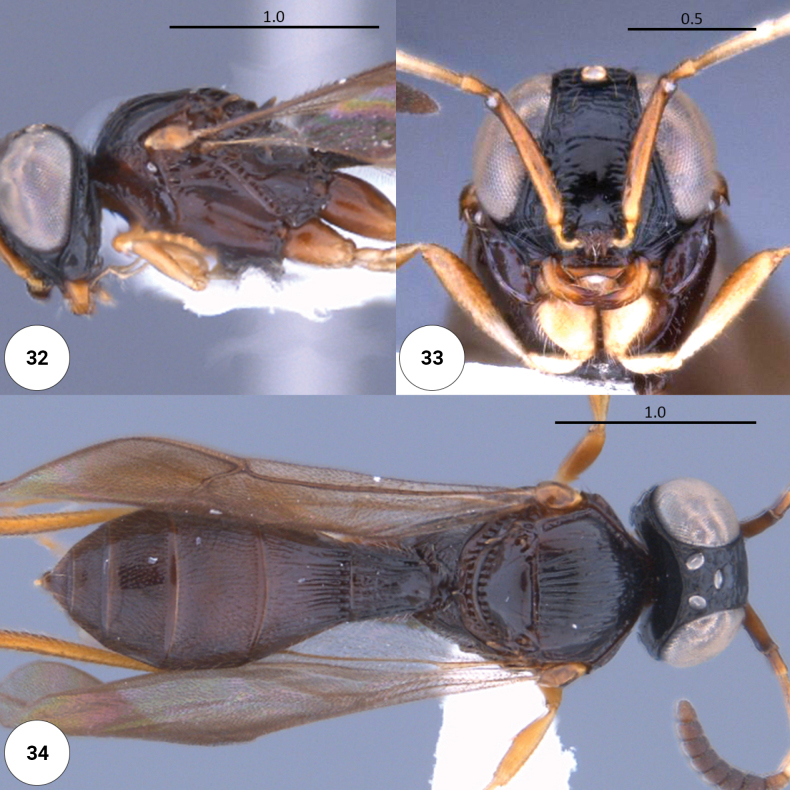
*Dichoteleasstriatus* (F) (CASENT 2043988) **32** head, mesosoma, lateral view **33** head, anteroventral view **34** dorsal habitus.

**Figures 35–37.**
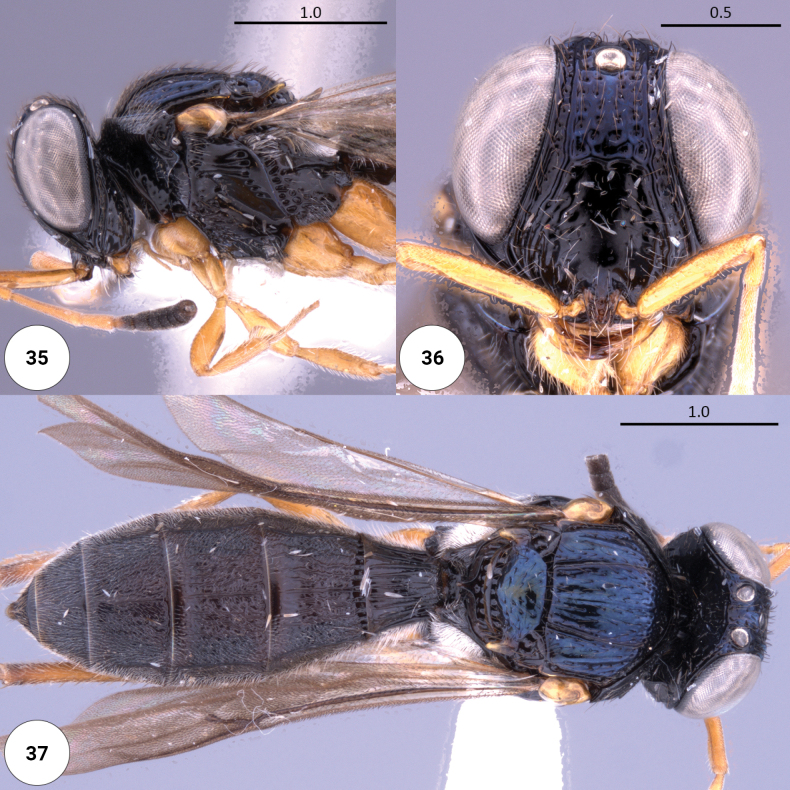
*Dichoteleassubcoeruleus* (F) (OSUC 0367538) **35** head, mesosoma, lateral view **36** head, anteroventral view **37** dorsal habitus.

##### Diagnosis.

This species can be distinguished by the presence of the median mesoscutual line and can be distinguished from *D.indicus* by the median carina on T1–T4.

##### Distribution.

Australia (Queensland)

##### Material examined.

***Holotype***, female: **Australia**: Queensland, Mossman, SAMA 32-00145 (SAMA). **Australia**: 13 females, 4 males, OSUC 367522, 367527–367542 (ANIC).

#### ﻿​ Dichoteleas umbra sp. nov.

Taxon classificationAnimaliaHymenopteraScelionidae

B9DCAA05-F236-5112-BA23-6F99F9367F34

https://zoobank.org/C86B76DC-5B02-4FDF-842F-60F2E98B4B06

Figs 38–40

##### Description.

Color of head: black. Hyperoccipital carina: present. Frontal depression: absent. Malar striae: present. Facial striae: present. Setation of eyes: absent. Sculpture of frons: smooth above interantennal prominence, areolate laterally. Setation of frons: sparsely setose throughout. Submedian carina: absent. Interantennal process: undifferentiated. Central keel: absent. Transverse pronotal carina: present. Color of pronotum: dark brown to black. Pronotal cervical sulcus: present. Mesepimeral sulcus: present. Sulcus along mesopleural carina: foveolate. Mesoscutal suprahumeral sulcus: absent. Mesoscutal humeral sulcus: present as an uninterrupted groove. Median mesoscutal line: absent. Color of mesoscutum: black with xanthic posterolateral corners. Sculpture of mesoscutum: punctate. Notaulus: complete. Visibility of notaulus: unobscured. Parapsidal line: present. Sculpture of mesoscutellum: smooth. Shape of axillular carinae in lateral view: without a posteroventral hooklike. Color of axillular carina: slighter lighter than mesoscutellum. Sculpture of T3–6: punctate. Median carina on T1–T4: absent.

##### Diagnosis.

*D.umbra* can be distinguished from *D.rugosus* by the xanthic posterolateral corners of the mesoscutum. This species differs from *D.hamatus* and *D.striatus* by the punctate mesoscutum.

##### Etymology.

The name *umbra* is from the Latin word for shadow or shade, referring to the dark color. This epithet is treated as a noun.

##### Distribution.

Tanzania (Uluguru Mts.).

##### Material examined.

***Holotype***, female: **Tanzania**: Uluguru Mts. Lupanga, East, 1300m; OSUC 875037 (CNCI). ***Paratypes*. Tanzania**: 1 female, 1 male, OSUC 875038-875039 (CNCI).

### ﻿Comments on undescribed specimens:

There were a few specimens that did not fit into these species descriptions. We have chosen to not formally describe them because all were male and only 1–2 specimens were available.

**Unknown 1**

**Material examined. Madagascar**: 1 male, CASENT2042862 (CAS).

Diagnosis. This specimen has an anteriorly produced IAP, similar to *D.indicus*, but it lacks a central keel and submedian carinae on the frons.

**Unknown 2**

**Material examined. India**: 2 males, OSUC 875042, OSUC 875043 (CNCI).

Diagnosis. These specimens have a curved carina in the shape of an inverted “U” present on the frons. It appears to join the facial striae anteriorly. Submedian carinae are present, and there is a blunt medial projection on the mesoscutellum. The specimens were collected in southern India, Tamil Nadu state (Coimbatore and the Anaimlai Hills).

**Figures 38–40.**
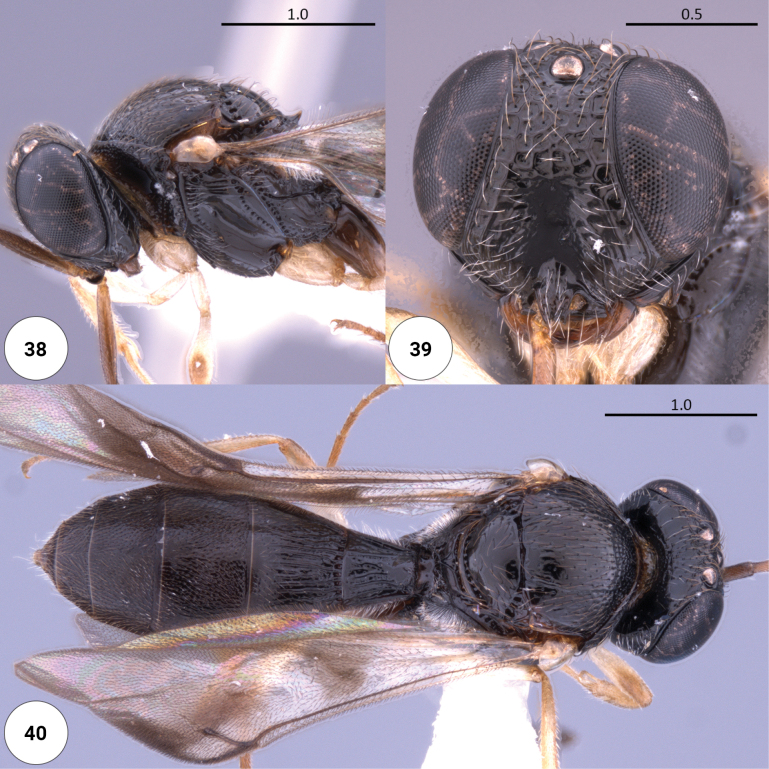
*Dichoteleasumbra* (F) (OSUC 875039) **38** head, mesosoma, lateral view **39** head, anteroventral view **40** dorsal habitus.

## Supplementary Material

XML Treatment for Dichoteleas

XML Treatment for Dichoteleas ambositrae

XML Treatment for Dichoteleas fulgidus

XML Treatment for Dichoteleas fuscus

XML Treatment for Dichoteleas hamatus

XML Treatment for Dichoteleas indicus

XML Treatment for Dichoteleas rubyae

XML Treatment for Dichoteleas rugosus

XML Treatment for Dichoteleas striatus

XML Treatment for Dichoteleas subcoeruleus

XML Treatment for Dichoteleas umbra

## ﻿Additional information

### Author ORCIDs

Simon Van Noort https://orcid.org/0000-0001-6930-9741

Norman F. Johnson https://orcid.org/0000-0003-1691-5187

### Data availability

All of the data that support the findings of this study are available in the main text.

## References

[B1] Austin AD Field SA (1997) The ovipositor system of scelionid and platygastrid wasps (Hymenoptera: Platygastroidea): comparative morphology and phylogenetic implications. Invertebrate Systematics 11(1): 1–87. 10.1071/IT95048

[B2] Bin F (1981) Definition of female antennal clava based on its plate sensilla in HymenopteraScelionidaeTelenominae. Redia (Firenze) 64: 245–261.

[B3] Brues CT (1908) Hymenoptera. Fam. Scelionidae. Genera Insectorum 80: 1–59. https://www.biodiversitylibrary.org/item/105248#page/202/mode/1up

[B4] Chen HY Lahey Z Talamas EJ Valreio AA Popovici OA Musetti L Klompen H Polaszek A Masner L Austin AD Johnson NF (2021) An integrated phylogenetic assessment of the parasitoid superfamily Platygastroidea (Hymenoptera: Proctotrupomorpha) results in a revised familial classification. Systematic Entomology 46(4): 1088–1113. 10.1111/syen.12511

[B5] Dodd AP (1913) Australian HymenopteraProctotrypoidea. No. 1. Transactions of the Royal Society of South Australia 37: 130–181.

[B6] Dodd AP (1926) New species of Australian Proctotrypoidea, with revisional notes. Proceedings of the Linnean Society of New South Wales 51: 369–381. 10.5281/zenodo.23802

[B7] Galloway ID (1976) the types of Australian species of the subfamily Scelioninae (Hymenoptera: Scelionidae). Queensland Journal of Agricultural and Animal Sciences 33(1): 83–114.

[B8] Galloway ID Austin AD (1984) Revision of the Scelioninae (Hymenoptera: Scelionidae) in Australia. Australian Journal of Zoology Supplementary Series 99: 1–138. 10.1071/AJZS099

[B9] Harris RA (1979) A glossary of surface sculpturing. California Department of Food and Agriculture, Bureau of Entomology. Occasional Papers in Entomology 28: 1–31.

[B10] Johnson NF (1992) Catalog of world Proctotrupoidea excluding Platygastridae. Memoirs of the American Entomological Institute 51: 1–825.

[B11] Kieffer JJ (1907) Beschreibung neuer im British Museum zu London aufbewahrter Proctotrypiden. Berliner Entomologische Zeitschrift 51: 279–302. 10.1002/mmnd.47919060306

[B12] Kieffer JJ (1908) Révision des Scelionidae (Hyménoptères). Annales de la Société Scientifique de Bruxelles 32: 111–250. https://www.biodiversitylibrary.org/item/157108#page/457/mode/1up

[B13] Kieffer JJ (1910) Hymenoptera. Fam. Scelionidae. Addenda et corrigenda. Genera Insectorum 80: 61–112. https://biostor.org/reference/132766

[B14] Kieffer JJ (1913) Proctotrypidae (3e partie). Species des Hyménoptères d’Europe et d’Algérie 11: 161–304. 10.3406/lsoc.1980.1236

[B15] Kieffer JJ (1926) Scelionidae. Das Tierreich Vol. 48. Walter de Gruyter & Co., Berlin, 885 pp.

[B16] Mani MS Sharma SK (1982) Proctotrupoidea (Hymenoptera) from India. A review. Oriental Insects 16(2): 135–258. 10.1080/00305316.1982.10434314

[B17] Masner L (1965) The types of Proctotrupoidea (Hymenoptera) in the British Museum (Natural History) and in the Hope Department of Entomology, Oxford. Bulletin of the British Museum (Natural History) Entomology Supplement 1: 1–154. 10.5962/p.97756

[B18] Masner L (1976) Revisionary notes and keys to world genera of Scelionidae (Hymenoptera: Proctotrupoidea). Memoirs of the Entomological Society of Canada 108(S97): 1–87. 10.4039/entm10897fv

[B19] Mayr E (1942) Systematics and the origin of species from the viewpoint of a zoologist. Columbia University Press, New York, USA.

[B20] Mikó I Vilhelmsen L Johnson NF Masner L Penzes Z (2007) Skeletomusculature of Scelionidae (Hymenoptera: Platygastroidea): head and mesosoma. Zootaxa 1571(1): 1–78. 10.11646/zootaxa.1571.1.1

[B21] Muesebeck CFW Walkley LM (1956) Type species of the genera and subgenera of parasitic wasps comprising the superfamily Proctotrupoidea (order Hymenoptera). Proceedings of the United States National Museum 105(3359): 319–419. 10.5479/si.00963801.3359.319

[B22] Rajmohana K (2006) Studies on Proctotrupoidea and Platygastroidea (Hymenoptera: Insecta) of Kerala. Memoirs of the Zoological Survey of India 21(1): 1–153. 10.11609/JoTT.ZPJ.1570.2506-13

[B23] Risbec J (1956) Proctotrupides Scelionini de Madagascar. Revue Française d`Entomologie 23: 244–264. [Hyménoptères]

[B24] Saraswat GG (1982) Some Indian Proctotrupoidea (Hymenoptera: Scelionidae). Records of the Zoological Survey of India 79(3–4): 343–358. 10.26515/rzsi/v79/i3-4/1981/161731

[B25] Szabó JB (1971) Eine neue Art der australischen Scelioniden, *Dichoteleaspappi* sp. n. (Hym., Proctotrupoidea). Folia Entomologica Hungarica 24: 313–318.

[B26] Wild AL (2004) Taxonomy and distribution of the Argentine ant, *Linepithemahumile* (Hymenoptera: Formicidae). Annals of the Entomological Society of America 97(6): 1204–1215. 10.1603/0013-8746(2004)097[1204:TADOTA]2.0.CO;2

